# Deep Learning-Based Object Detection, Localisation and Tracking for Smart Wheelchair Healthcare Mobility

**DOI:** 10.3390/ijerph18010091

**Published:** 2020-12-24

**Authors:** Louis Lecrosnier, Redouane Khemmar, Nicolas Ragot, Benoit Decoux, Romain Rossi, Naceur Kefi, Jean-Yves Ertaud

**Affiliations:** 1 École Supérieure d’Ingénieurs en Génie Électrique, 76800 Saint-Étienne-du-Rouvay, France; benoit.decoux@esigelec.fr (B.D.); romain.rossi@esigelec.fr (R.R.); Jean-Yves.Ertaud@esigelec.fr (J.-Y.E.); 2 SUP’COM: École Supérieure des Communications de Tunis, Carthage University, Aryanah 2080, Tunis; naceur.kefi@supcom.tn

**Keywords:** object detection, tracking, distance estimation, smart mobility, object localization, distance measurement, deep learning, computer vision, semantic map

## Abstract

This paper deals with the development of an Advanced Driver Assistance System (ADAS) for a smart electric wheelchair in order to improve the autonomy of disabled people. Our use case, built from a formal clinical study, is based on the detection, depth estimation, localization and tracking of objects in wheelchair’s indoor environment, namely: door and door handles. The aim of this work is to provide a perception layer to the wheelchair, enabling this way the detection of these keypoints in its immediate surrounding, and constructing of a short lifespan semantic map. Firstly, we present an adaptation of the YOLOv3 object detection algorithm to our use case. Then, we present our depth estimation approach using an Intel RealSense camera. Finally, as a third and last step of our approach, we present our 3D object tracking approach based on the SORT algorithm. In order to validate all the developments, we have carried out different experiments in a controlled indoor environment. Detection, distance estimation and object tracking are experimented using our own dataset, which includes doors and door handles.

## 1. Introduction

Object detection, recognition, localization and tracking are very important tasks in mobile robotics and computer vision applications. These processes are achieved through the use of different measurement sensors (camera, LIDAR, RADAR, etc.) and algorithms (filtering, object detection, pattern recognition, feature extraction, segmentation, classification, etc.). The work presented in this paper focuses on object detection, localization and tracking for a smart wheelchair. Through the ADAPT (“Assistive Devices for empowering disAbled People through robotic Technologies”, http://adapt-project.com) project, the detection and classification of objects which are specific to the wheelchair indoor environment, are a key issue for the safety navigation of the wheelchair. More specifically, these objects are doors and door handles. Our contribution aims at developing a system able to detect specific objects, track them and measure their depth, for our smart wheelchair. For that objective, we use a combination of a deep-learning algorithm for object detection and various techniques for the other tasks. For object detection, we developed a specific dataset, and validated the complete approach through the ESIGELEC Autonomous Navigation Laboratory (ANL). Our objective is not only to develop three key steps in wheelchair environment perception, namely: detection, localization and tracking, but also to implement them on the wheelchair. For that matter, we intend on taking into account the embedded system constraints such as real time and CPU/GPU computing resources, and memory space, in order to increase the wheelchair autonomy in terms of semi-autonomous navigation. The remainder of this paper is organized as follows. Section 1 introduces the paper. In Section 2, we present the state-of-the-art of object detection, tracking and distance estimation dealing with deep learning approaches, and semantic map generation. The architecture of the smart wheelchair platform is presented in Section 3. Our approach related to object detection, depth estimation and tracking implemented as a full embedded system is then detailed in Section 4. Section 5 describes the experimental results and, finally, Section 6 concludes this article.

## 2. State of the Art

### 2.1. Object Detection

Object detection methods based on deep-learning are among those giving the best performances on all methods. They can be divided into two main categories: 1. one-stage methods, which perform the object localization and object classification in a single network, and 2. two-stage methods, which have two separated networks for localization and classification [1]. In the first category, we find the YOLOv3 (You Only Look once) algorithm [2], in which classification is made on a predefined number of bounding boxes of given sizes at specific layers. Among the one-stage state-of-the-art methods, we can also cite Single-Shot Detector (SSD) [3]. Like YOLO, SSD combines methods for locating and classifying Regions of Interest (ROI), thus avoiding resampling of pixels and features extracted from the image for each bounding box. To perform feature extraction, SSD uses the VGG-16 [4] architecture for the first layers of the network, but can be used with any other backbone. Subsequent layers are progressively reduced in size to allow multi-scale processing, thus allowing detection of small objects. Each of these layers produces a set of detection predictions. Methods of the second category consist of two modules present in a Convolutional Neural Network (CNN). The first module returns the coordinates of ROIs where objects (whatever their nature) are likely to be present in the image. The second module is dedicated to classification, providing class predictions for the proposed regions. Methods of this category include the Fast R-CNN [5], Faster R-CNN [6] and Mask R-CNN [7]) algorithms. One-stage approaches are generally faster than two-stage ones, with similar performance [2]. In our application, real-time performance is a critical point, which makes us prefer a one-shot method. Furthermore, our application is based on spatio-temporal data (through video sequences) [1], which is redundant information over time, so imprecise detection of small objects in all images is not a critical problem.

### 2.2. Distance Measurement and Depth Estimation

Visual environment perception is a key component in the development of smart mobility. This includes object detection, tracking and localization, but also recognition, in real-time. In the case of disabled people using a wheelchair, some specific objects are of particular interest: doors, door handles, light switches, etc. Distance measurement from objects can be based on various sensors: ultrasound sensors, lasers, radars, infrared sensors, cameras, etc. Some of those solutions are costly and can hinder development of such systems. Other solutions include image processing, which can be monocular or stereoscopic. Many of those methods are based on deep-learning algorithms. Overviews on deep-learning based monocular and stereoscopic algorithms can be found in [8,9] (respectively).

For estimation of distance, several approaches are used within the literature and are divided into two main families: monocular and stereoscopic approaches. In monocular approaches, if the ground truth for the depth information is available, is it possible to directly train a CNN to output a depth image. Most of the CNN models for depth estimation have an encoder–decoder structure, like the CNN used in semantic segmentation [10,11]. These models have some main problems like the lack of full resolution at the output of the network, due to the bottleneck at the junction between the encoder and decoder parts. Another solution is to use an unsupervised approach to estimate depth from monocular images. In [12], the authors present an unsupervised deep neural network for single image depth estimation. The model is trained on stereo images but infers disparity maps from monocular images. Instead of using ground-truth depth data, the model exploits the fact that depth information can be trivially computed from a calibrated stereo camera setup using disparity images. In [13], a Multi-Scale Local Planar Guidance model is presented for depth estimation. In the same category, DenseDepth [14] has an encoder part which is initialized with meaningful weights and based on a pretrained DenseNet [15], and then trained with NYU Depth v2 [16], Unreal-1K [14] and KITTI datasets. The approach gives a depth map that capture object boundaries more faithfully, thanks to a specific loss function penalizing high-frequency distortion.

In this work, we chose to rely directly on a depth sensor instead of using the depth estimation algorithm (that we have used in a previous work [17]), in order to alleviate the computational cost. Hence, we investigate the use of two cameras for the task of distance measurement, as it is one of the less costly sensors among the ones cited above.

### 2.3. Tracking Methods

For the benchmarking of object-tracking algorithms, a library of source-code, an annotated dataset and all the tracking results are available online [18]. Here, we focus on realtime algorithms, as it is a strong constraint for our application.

In [19], single-target tracking is performed at 100 Frames Per Second (FPS) on a GPU by means of Deep Regression Networks (DRN), after training with a set of labeled videos and images. A search region from the current frame and a target from the previous frame are given as input to a 2-branch CNN with a simple architecture. Another algorithm called SORT (for Simple Online Realtime Tracking) [20] is also aimed at running in realtime, with a FPS of 260 on a single-core CPU. It is based on Faster R-CNN and uses Kalman filter for prediction of target positions in future frames. This model has been completed by an appearance model to improve its performance, specifically by allowing longer periods of occlusion, then reducing the number of identity switches [21]. ROLO (for recurrent-YOLO) [22] is another solution which consists in using an object-detection algorithm like YOLO to which a recurrent layer based on Long Short-Term Memory (LSTM) units [23] is added, thus bringing tracking ability to the model.

### 2.4. Semantic Mapping

Once detected in images, semantic information can be associated with a 2D map of the environment by means of odometry information. This semantic information can be available pixel-wise (“semantic segmentation”) or from a bounding box (detection). This allows augmentation of the map towards a 3D reconstruction of the environment. This operation is important in robotics and visual navigation.

In [24], the output of a semi-dense Simultaneous Localisation And Mapping (SLAM) system based on a video stream from a monocular camera, is combined with the output of an encoder-decoder CNN providing 2D semantic segmentation on selected keyframes, to generate a semantic 3D map. In [25], a similar process is used, but semantic segmentation is applied on all the images from the video stream and depth information from a RGB-D camera is integrated to the process. In [26], an unsupervised geometric segmentation of objects is obtained from depth frames of a RGB-D camera, and combined with semantic object instance predictions from the Mask R-CNN algorithm, to generate the mapping. In [27], a CNN-based object detector (YOLO) is used to detect some specific objects in indoors environments (doors, fire extinguishers, benches, water fountains) along with a 3D model-based segmentation technique to perform instance semantic segmentation.

### 2.5. Datasets

For object detection and tracking, we need one or more datasets for the learning of the CNNs. We are primarily interested by datasets that contain classes directly related to our application (i.e., doors and door handles). Datasets that do not include these objects can nonetheless be useful to pre-train the first layers of a CNN, as those layers learn features low level image features.

**Figure 1:**
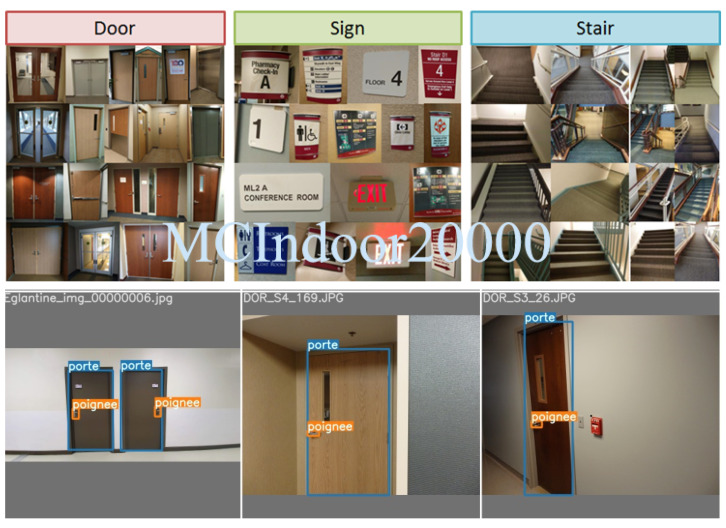
MCIndoor2000 dataset image sample (**top row**), custom dataset (**bottom row**).

Various datasets are freely available for object detection. ImageNet [28] includes 14,000,000 object instances from 1000 classes [29]. In Open Images [30], a community dataset with over 9,000,000 images, objects are labeled with ROI. It consists of 7881 different classes [31] very varied and constantly expanding. COCO [32] includes 1,500,000 images for which the objects from 80 classes are labeled and categorized. COCO-stuff [33], an extended version of COCO with more than 1,800,000 image, includes 181 classes [34]. PASCAL VOC [35] is dedicated to pedestrian detection with 500,000 tagged images. It includes 181 classes of 20 different objects. CIFAR-100 [36] includes 60,000 images with objects from 100 classes. INRIA has developped its own dataset dedicated to pedestrian detection. Caltech dataset is also dedicated to the detection of people and pedestrians. Overall, these datasets are relatively rich concerning the most commonly detected objects (pedestrians, cars, buses, bicycles, etc.). The COCO dataset is also the reference in terms of object detection and is ideal for comparing different object detection models. The latter offers different databases adapted to the training phases and to the test of neural networks. YOLOv3, for example, uses ImageNet to train the first 53 layers of its network and establish the reference databases. This dataset being very dense, it is a good choice to pre-train YOLOv3. This model then uses other databases to perform detection and classification, such as COCO for example. For more specific applications, MCIndoor20000 dataset [37] includes more than 20,000 digital images from three different indoor object categories, including doors, stairs, and hospital signs. Figure 1 shows some examples of MCIndoor2000 dataset images including doors and handles, as well as a sample of our custom ESIGELEC dataset.

The wheelchair is supposed to navigate indoors as well outdoors. For this reason, the datasets dedicated to the autonomous vehicle are also interesting to take into account. In KITTI [38], the authors present one of the widely used dataset in road environment for autonomous driving research. KITTI is a calibrated, synchronized, and timestamped autonomous driving dataset. The KITTI dataset was collected through instrumented vehicle by different kinds of sensors such as: color and grayscale stereo cameras, a velodyne 3D laser scanner and a high precision GPS/IMU navigation system. The platform contains real-world traffic situations with both static and dynamic objects [38]. NuTonomy scenes (called NUScenes [39]) is a multimodal dataset for automotive driving applications, with information from several kind of sensors such as: 6 cameras, 5 radars and 1 LiDAR. It is fully annotated and comprises 1000 scenes, 3D bounding boxes for 23 classes and 8 attributes. It has 100× as many images than the pioneering KITTI dataset. It also contains dataset analysis as well as baselines for LiDAR and image based detection and tracking [39].

## 3. Smart Wheelchair Platform Architecture

### 3.1. Hardware Architecture

The robotic electric wheelchair of the IRSEEM laboratory is an Invacare, Bora model, from which all the proprietary electronics have been removed and replaced by our own elements: 1. An on-board computer with 8 GB RAM and 250 GB SSD, running on Linux Ubuntu 16.04 LTS; 2. A Roboteq engine driver; 3. An Xbox joystick instead of the original one, which can be connected to the wheelchair by an USB or a bluetooth connection to manually control the wheelchair; 4. A Wi-Fi router to get a wireless LAN on the wheelchair; 5. An embedded Human-Machine Interface (HMI) with a touch screen; 6. The sensing part is composed of an Intel RealSense D435 camera and an Realsense T265 camera. The D435 camera provides color and depth images, whereas the T265 camera provides fisheye images and odometry data in the form of position (cartesian coordinates) and orientation (quaternions). In addition, an on-board computer is used, with Ubuntu 16.04 as operating system and 8 GB RAM and 250 GB SSD, for all on-board processing. The interaction between the wheelchair and the user is done via a touch screen which is connected to the on-board computer via an HDMI connection, or using the Wi-Fi connection. A Roboteq engine driver is used to control the engines.

**Figure 2:**
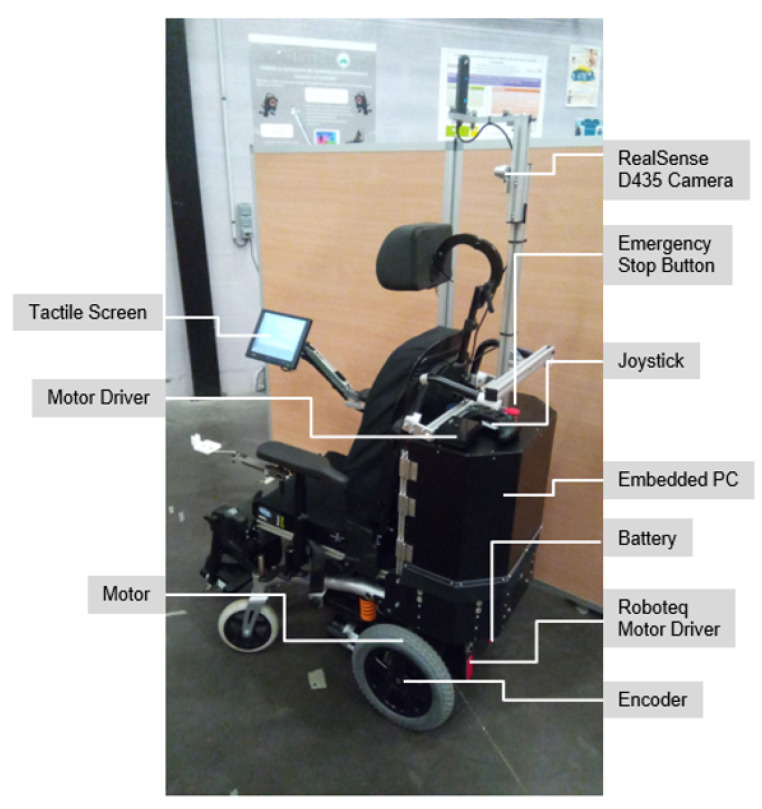
IRSEEM Electric Wheelchair. Note: the T265 RealSense camera used for odometry was not mounted for this photo. For the experiments described in the text, it was placed near the D435 camera.

The hardware description of the wheelchair is shown in Figure 2.

In our project, the wheelchair should be autonomous in decisions. Because of the deep learning algorithm involved in the object detection process, we needed a GPU-capable embedded computer. For this reason, we have implemented the entire software developments onto an Nvidia Jetson TX2 board. This board offers several advantages: GPU computing power for our deep learning algorithm, software interface fully compatible with image processing and computer vision libraries. Using a Wi-Fi or ethernet connection, the Jetson TX2 can exchange data with the embedded computer, perform deep-learning-based detection, and send back the results at high speed.

### 3.2. Software Architecture

The software architecture of the processing chain consists of 3 parts:

- Image acquisition: two kind of images are acquired through the D435 camera: RGB (color) and depth images;
- Object detection and depth estimation: objects (doors, handles) are detected using the color images provided by the D435 camera. Distance estimation is carried out using the depth images, and provides distance measurements to be associated with the detections;
- Tracking: using a classified object and an associated depth, the position of the 3D object is added to the semantic map using the odometry data provided by the T265 camera.

**Figure 3:**
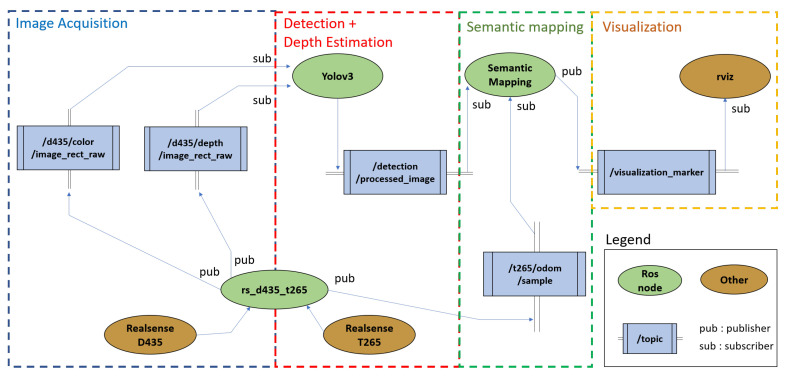
ROS Software Architecture.

The wheelchair software is fully developed under the Robotic Operating System (ROS) robotic middleware [40]. Because ROS is a multi-platform framework, portability between desktop and embedded platform was completely transparent, and required no code refactor. The basic principle of ROS as a robotic Operating System is to run in parallel a large number of executable that must be able to exchange information synchronously or asynchronously [41]. In our wheelchair architecture, ROS allows to poll, at a defined frequency, the wheelchair sensors (perception cameras, localisation camera, encoders odometer), retrieve these data, pre-process them, apply deep learning algorithms (sensors calibration, image acquisition, object detection, depth estimation and distance measurement, wheelchair localization, semantic mapping, and visualization) and finally control the wheelchair motors (navigation algorithms) in return. All this processes are carried out continuously and in parallel. On the other hand, ROS must manage the competition between all these processes in order to ensure efficient access to wheelchair’s resources. Figure 3 shows the wheelchair’s ROS software architecture.

## 4. Object Detection and Distance Measurement

### 4.1. Object Detection

To perform object detection, we have used the powerful YOLOv3 neural network.

**Figure 4:**
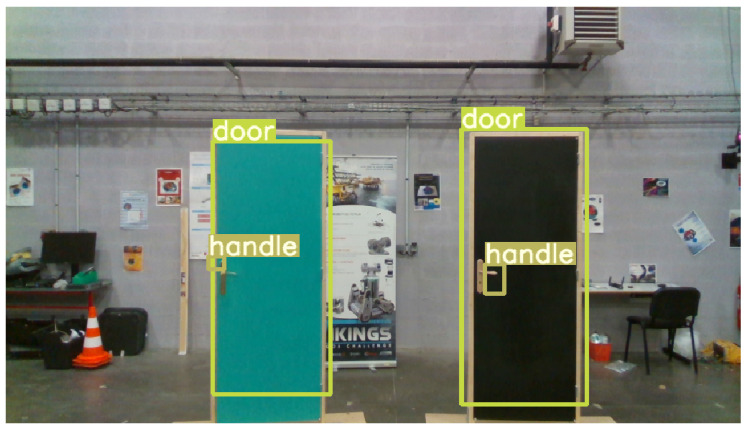
Example of objects detection in wheelchair indoor environment including doors and handles.

For this project, the keypoints we detect are doors ans door handles. Since these classes are under-represented in the YOLOv3 training dataset (i.e., ImageNet [28]), we have composed a custom dataset to perform the network training. We have extracted 755 door images from the MCIndoor20000 dataset [37], consisting of tagged images containing various wheelchair indoor objects. As we could not find an open dataset with sufficient representation door handles, we have developed our own dataset, consisting of 1885 images, which we combined with door images from the MCIndoor20000 dataset. We supervised the labeling of 2640 images from two combined datasets using a semi-automatic labeling tool we developed. This tool is a simple drag-to-select python script, that allowed at first a manual annotation of about 1000 object per class. We then used these annotated images to retrain YOLOv3. From this retrained model, we were able to classify a larger dataset. We selected the properly labeled images from this extended dataset, that we again used to further refine the model. We finally proceeded to fine tune YOLOv3 on the required classes. For this transfer learning process, we trained only the neural network classification layers. Figure 4 shows some qualitative results of the detection process after re-training YOLOv3 on the recognition of doors, and door handles.

### 4.2. Depth Estimation

Once the detection step has been performed, a distance is associated to each extracted object. The RealSense D435 camera can provide a depth image with an accuracy of around 10% for distances between 0.10 m and 20 m. Nevertheless, there is a difference in field of view between the pair of stereoscopic infrared cameras providing depth images, and the color sensor. It is therefore necessary to match the color and infrared images. This is done using the manufacturer’s data. At the end of this step, the distance values for each pixel are extracted from the ROI returned by YOLOv3. We then compute the median distance from all the distance values available for this sub-image. Using this method, we are able to discard missing depth values, i.e., null distance, and outliers, i.e., distance values with high error. Hence, we obtain a more accurate depth value than if we simply considered the depth value corresponding to the pixel in the middle of the detection bounding box. Moreover, because the objects we detect are either planar or of small size, the median depth value of the objects bounding box matches its 3D center. Figure 4 illustrates the result of the ROI extraction process and the assignment of associated depth information.

### 4.3. Object Tracking

**Figure 5:**
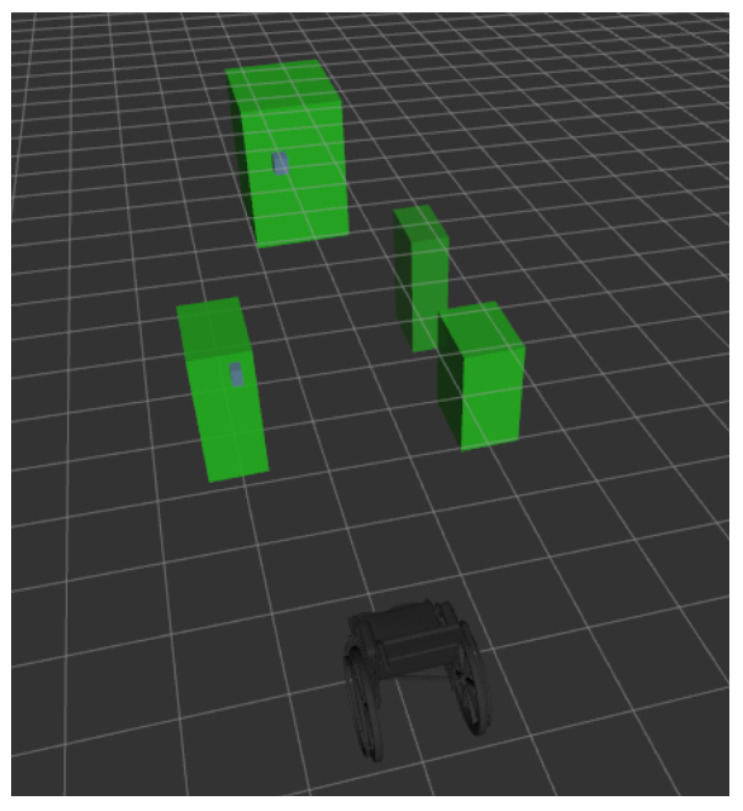
A 3D local semantic map from a hallway. Bottom image: the wheelchair. Top image: objects (doors) in the environment.

Using the combination of position and depth associated with each item extracted by the detection algorithm, we rely on the SORT (Simple Online and Realtime Tracking) algorithm [21] to track the different objects in the scene while the wheelchair is moving. On a video stream, multiple objects (doors and door handles) are detected and assigned a given distance. SORT inspects the detected objects, and determines if a given object is newly seen, or if the movement of the object is a consequence of the wheelchair’s movements. This algorithm is based on a Kalman filter and provides a unique identification number to each new detected object. SORT keeps track of several objects simultaneously and filters the positions of noisy objects associated with moving boundary boxes. Finally, we use odometric data from T265 RealSense camera to estimate the wheelchair displacement. We combine this data with the position of the object in order to visualize a 3D semantic map of the environment containing the detected and tracked objects, as shown in Figure 5.

In order to validate these algorithms, we created out an experimental dataset in the ANL IRSEEM laboratory. A set of four doors with different colours and handle shapes were placed in the coverage area of a motion capture Vicon system. Once equipped with reflective markers, the doors and wheelchair were located by a Vicon system providing their position and orientation with millimetric precision at a frequency of 100 Hz [42]. This way, we obtain a high rate ground truth distance between the wheelchair and the doors and handles.

**Figure 6:**
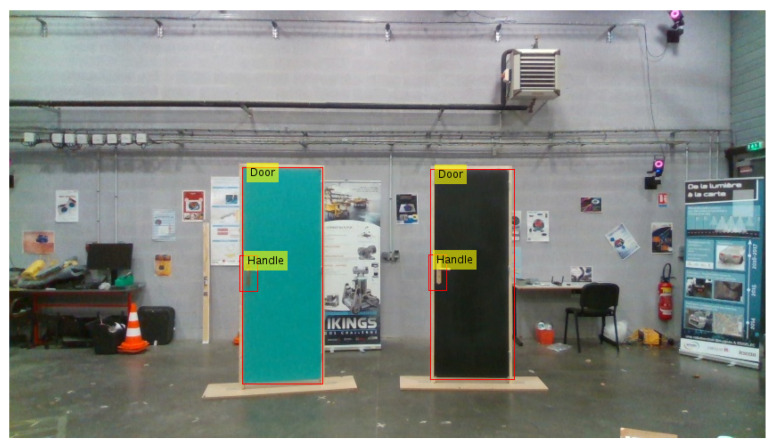
Arrangement of doors in the validation dataset. The ground truth bounding boxes are displayed in red with the associated label overlaid in yellow. In the **top-right** of figure, we can see a part of the Vicon motion capture system within cameras network.

The different doors have been placed along an arc of a circle (see Figure 6), with the aim of evaluating the door detection when multiple elements are present simultaneously in the images. In this experiment, ADAPT’s wheelchair moves through the scene and, by changing orientation, finds itself several times facing the different doors. The distance measurements between the doors, handles, and the wheelchair is then compared to the ground truth provided by the Vicon system.

Figure 9 presents these results. For a total of 804 doors detections, the actual distance and the distance estimated by the D435 RealSense camera, as well as the difference between these two values, are observed. Table 2 provides the numerical results of this experiment. We observe a mean error of 18.1 cm, corresponding to 3.8% error on the estimation of the objects distance which is a good result regarding the indoor environment constraints of the wheelchair. These values are below the data provided by the manufacturer. Figure 5 illustrates some examples of semantic maps obtained under our ROSBAG dataset. The SORT algorithm allows us to keep track of all the detected objects in the scene. By applying a 3D clustering algorithm, we determine the position of the labeled objects, and update the semantic map.

## 5. Experimental Results

For our experiments, we have arranged our ANL in such a way to have an indoor environment similar to the home of people who have reduced mobility, with rooms, corridors, doors, handles, etc. A video example of our platform is provided online (https://youtu.be/ieTtsGvShz4).

### 5.1. Object Detection Evaluation

Before presenting our experiments for object detection and distance measurement, we need to define the different metrics that we use in the evaluation process. We do not use the different metrics known in the state-of-the-art such as relative error, squared relative error, root mean squared error or logarithmic root mean squared error. As our object detection is based on classification, we use three metrics: IoU, precision and recall. For the precision, the Positive Predictive Value (*PPV*) is presented in Equation (1).

(1) $$PPV=\frac{TP}{TP+FP}$$

where TP is true positive, and FP is false positive.

The Recall value defined as the True Positive Rate (*TPR*) is presented in Equation (2).

(2) $$TPR=\frac{TP}{TP+FN}$$

where FN is false negative.

**Figure 7:**
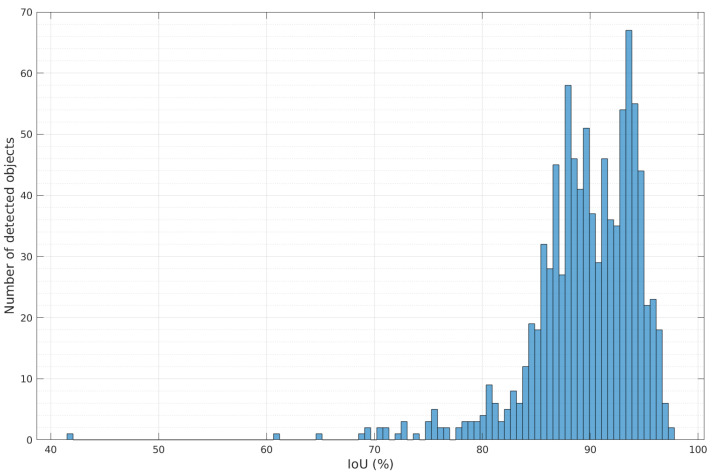
Distribution of IoU values for the detected doors.

In order to label large objects correctly, we rely on the IoU metric. We chose a threshold of 0.50 under which we consider a detection as incorrect. While suited for larger objects such as doors, the IoU becomes less relevant for small objects. At a distance of 5 m, the bounding box of handles represents only 2% of the entire image. In that case, even a very low IoU value can correspond to a correct detection. Figure 7 presents the IoU distribution of the detected doors. We note here that 89% of the IoU values are above the 0.5 threshold, implying a high number of correct detections. This is consistent with results shown on Table 1, which provides the precision and recall rates for the doors and handles detection process, as well as the average, median and standard deviation for the doors detection.

In Table 1, we observe a precision of 85% and 90% for the classification of handles and doors respectively, with a respective recall rate of 0.29% and 0.80% on a set of 866 images. It is likely that the low recall rate of handles is due to the lack of diversity in our dataset. The recent updates of YOLO does provide advanced tools for automatic data augmentation, which were not available at the time of our experimentation. Data augmentation and gathering of a more extensive dataset will be part of our future work, and should improve the current results.

For this purpose, we plan on developing our own data collection system for the dataset, materialized by a D435 RealSense camera instrumented on the wheelchair as shown in Figure 1. We will introduce a protocol for images collection in indoor environment that will take into account not only the ANL as a controlled environment, but also some corridors (with doors and handles) inside the IRSEEM building. In addition, we also plan to increase our dataset diversity using deep learning-based data augmentation built within YOLOv5. Data augmentation performs transformations (i.e., rotations, adding noise, mixing subsets of images, etc.) on the base training data to expose the model to a wider range of semantic variation than the base training set [43].

When tested on our desktop computer which is equipped with a Nvidia GTX 980 GPU, we are able to process the image flow of the camera (30 FPS) in real-time. When tested on the Jetson TX2 GPU, we get a framerate of 5.8 FPS at the same 640×480 resolution, which is still suitable for our use case in which the wheelchair moves at at most 1 m per second. We did consider resizing the images when using the Jetson TX2. We do tend, in this case, to have a faster inference. However, due to the size of some of the objects we want to detect (i.e., handles), lowering the resolution leads to a degradation in term of detection performance, especially for far and small objects. We explain this phenomenon by the fact that the downsampling drastically reduces the details of these objects, which complicates the work of YOLOv3.

### 5.2. Distance Estimation Evaluation

**Figure 8:**
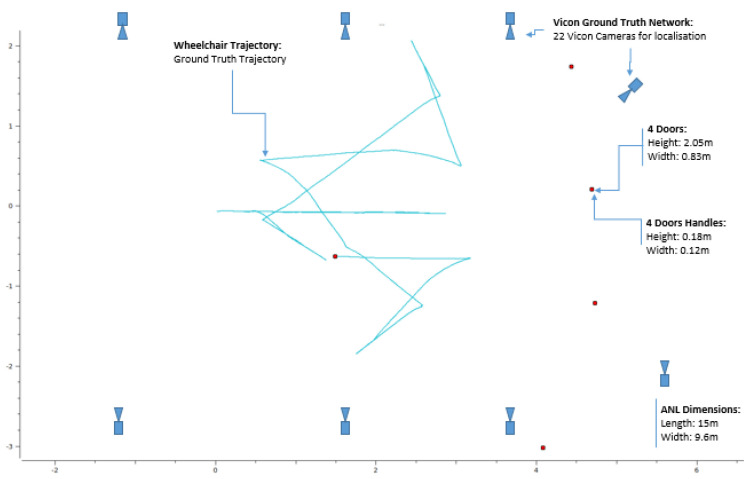
ANL environment including: Wheelchair, doors, doors handles, and Vicon cameras ground truth system.

For the distance evaluation between the camera (which is mounted on the wheelchair) and the different objects in the indoor environment, we used in previous works different CNN models dedicated to depth estimation like Monodepth [12], Monodepth2 [1,44], and MadNET [17,45]. In this paper, we have carried out distance measurements by directly using the RealSense D435 camera (without any deep learning model) because of embedded-system constraints related to the wheelchair such as: not enough GPU computational power on the Jetson TX2 board, not enough space memory, and the distance estimation of object which should be done in real time. This is why in this section we will present only the distance measurement by camera. We compare the distance obtained through the camera with the ground truth data given by the Vicon motion capture system available in our ANL laboratory. We have arranged the ANL in such a way that we have an indoor environment like indoor home of disabled people with reduced mobility, with rooms, corridors, doors, handles, etc. Figure 8 shows one example of ANL navigation environment for the wheelchair with doors, handles, and the Vicon ground truth system.

**Figure 9:**
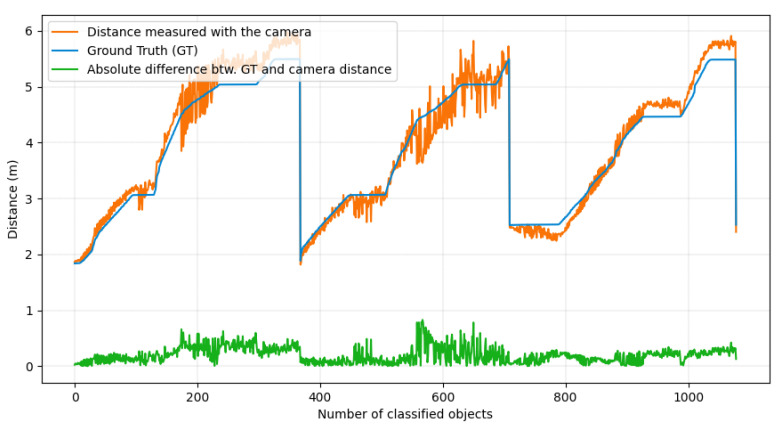
Measured distance between correctly classified objects and D435 RealSense camera (orange), ground truth distance (blue), absolute value of the difference between the ground truth distance and the distance estimated by the camera (green). The detected objects are sorted by class, then detection order.

Figure 9 shows the measured distances between objects and the camera, as well as the absolute distance measurement error, in comparison with millimetric ground truth from the Vicon motion capture system.

**Figure 10:**
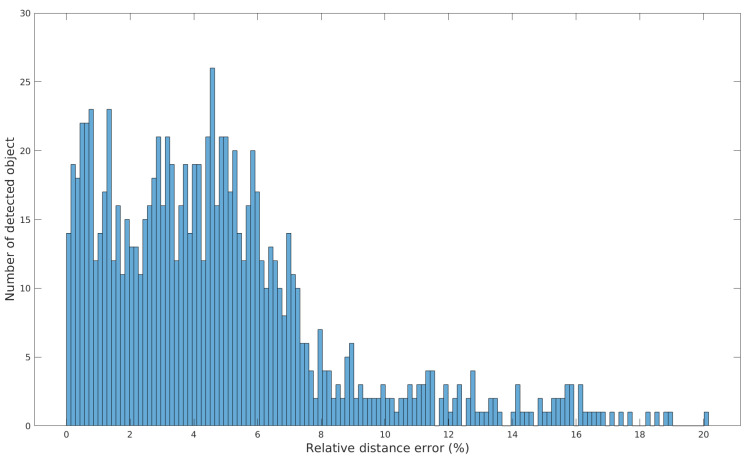
Distribution of the relative distance measurement error.

The distribution of the relative distance measurement error is shown in Figure 10.

In Table 2, we present the different results of the depth estimation obtained on our own dataset. Because of the median filter applied to the distance measurements of the Realsense D435 camera, we obtain a significantly lower error than that indicated in the sensor datasheet, with a 3.8% average error instead of the 10% error stated by the manufacturer.

In addition to the data provided in Figure 9 and Table 2, we observe on Figure 10 that most of the relative distance measurement error is distributed between 0% and 8%, centered around 3.8%, and with a standard deviation of 2.6%. This distribution is inherent to detection inaccuracies.

In our algorithm, the depth measurement of an object is the median value of the depth sub-image corresponding to the bounding box of the detected object. This median calculation acts as a spatial filter, but is dependent on the sensor measurement output.

**Figure 11:**
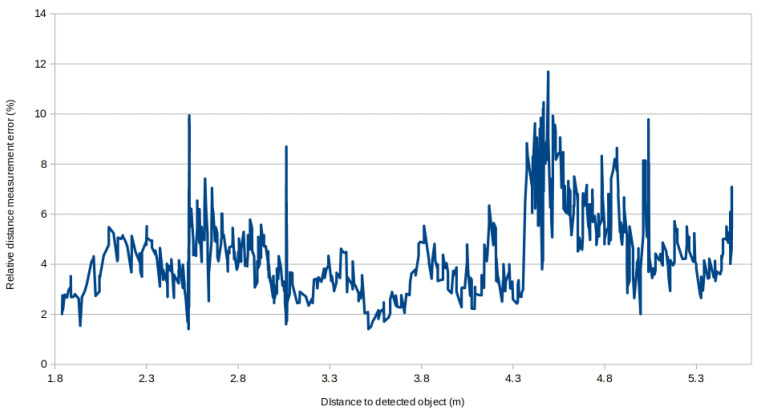
Distribution of the relative distance measurement error, relative to the distance of the detected objects, sliding 5 points average.

We can see on Figure 11 the representation of the 5 points sliding average of the distance measurement error rate, relative to the distance of the measured objects. This figure shows that the relative distance measurement error varies greatly, from less than 2% to over 12%, independently of the distance to the detected object.

When the detection algorithm outputs a bounding box poorly centered on the ground truth, i.e., The IoU has a low value, the median depth value can fall outside the object. In our dataset case, the doors are placed in the middle of our lab, instead of onto a wall. For this reason, when the IoU is too low, the depth measurement algorithm can returns a value corresponding to the wall behind the detected object. This is especially true for small objects such as handles. This explains the peaks noticeable at 2.5 and 4.5 m on Figure 11. For a future work, we plan on increasing the depth measurement precision by applying a temporal filter, in combination to the spatial filter, in an attempt to further refine the depth measurement accuracy.

## 6. Conclusions and Future Work

In this paper, we have presented an object detection, depth estimation, location and tracking system for wheelchair healthcare smart mobility, specifically for indoor environment, based on deep learning for object detection (YOLOv3), and various techniques and algorithms. We have measured the distance estimation error with the detected objects, by comparing the distance measured by the camera and the ground truth data given by the Vicon motion capture system. To validate the whole developments, our models have been re-trained using two datasets: the open MCIndoor20000 dataset and our own ESIGELEC dataset. By using our re-trained and fine-tuned model, our implementation offers good performance in inference on the wheelchair indoor environment. All developments are integrated on the platform via an Nvidia Jetson TX2 board as the main embedded computer for all algorithms (object detection, distance estimation, and tracking). Among the added values of this work, we can mention the integration of an object detection and localization chain on the wheelchair while optimizing our algorithms so that they are compatible with real-time constraints. The use of two cameras, one for distance measurement and the other for localization of the wheelchair, also represents a strong added value. Finally, this work allowed us to validate our own database (866 images). As a future work, and in order to finalize our smart healthcare wheelchair platform, we plan on improving the quality of the dataset by extending out image dataset for the indoor environment, including more classes such as doors, handles, switches, corridors, etc. Updating the object detection neural network to a more recent version, i.e., Yolov5, and refining the training using data augmentation should also increase the performance in term of speed, precision and recall. The next step after object detection, localisation, and tracking chain is semantic segmentation in order to carry out a complete scene understanding. For this, we plan to develop a virtual dataset with which we will enable the training of a semantic segmentation deep learning model. The wheelchair needs to recognize its environment with high accuracy in order to execute different scenarios such as: (i) the system detects a door and automatically controls the wheelchair to navigate to it, and then detects the handle on the door to allow opening; (ii) the system detects a light switch and automatically drives the wheelchair to it to alow light activation, etc.

## Figures and Tables

**Table 1:** Evaluation of the object detection.

Class	Mean IOU	Median IOU	Std. Dev. IOU	Precision (%)	Recall (%)
door	0.89	0.89	0.05	0.90	0.80
handle	NA	NA	NA	0.85	0.29

**Table 2:** Evaluation of depth estimation on 960 combined doosr and handles detection with an average distance of 3.75 m to the detected objects.

Depth Estimation Error	
Median (cm)	15.6
Average (cm)	18.1
Standard deviation (cm)	13.5
Median (%)	3.2
Average (%)	3.8
Standard deviation (%)	2.6

## Abbreviations

The following abbreviations are used in this manuscript:

ADAS	Advanced Driver Assistance System
ANL	Autonomous Navigation Laboratory
CNN	Convolutional Neural Network
COCO	Common Object in Context
YOLOv3	You Only Look Once
ROI	Region Of Interest
DRN	Deep Regression Networks
FPS	Frame Per Seconde
SORT	Simple Online Realtime Tracking
EKF	Extended Kalman Filter
LSTM	Long Short-Term Memory
SLAM	Simultaneous Localisation and Mapping
NUScenes	NuTonomy Scenes
HMI	Human Machine Interface
IoU	Intersection over Union
ROS	Robotic Operating System
PPV	Positive Predictive Value
TPR	True Positive Rate
SORT	Simple Online and Realtime Tracking
INRIA	Institut National de Recherche en Sciences et Technologies du Numérique
